# Protein-Based Blood Substitutes: Recent Attempts at Controlling Pro-Oxidant Reactivity With and Beyond Hemoglobin

**DOI:** 10.3390/ph6070867

**Published:** 2013-07-04

**Authors:** Violeta-Florina Scurtu, Augustin C. Moţ, Radu Silaghi-Dumitrescu

**Affiliations:** Department of Chemistry, “Babeş-Bolyai” University, 1 Mihail Kogalniceanu Str., Cluj-Napoca RO-400084, Romania; E-Mails: florinadeac@chem.ubbcluj.ro (V.-F.S.); augustinmot@chem.ubbcluj.ro (A.C.M.)

**Keywords:** hemoglobin, blood substitute, oxidative stress

## Abstract

Reviewed here are recent attempts to produce protein-based artificial oxygen carriers (“blood substitutes”). Most of these involve chemical or physical modifications on hemoglobin, although a recent line of research using hemerythrin instead of hemoglobin is also described. The focus is set on the extent to which these modifications alter the redox reactivity of the proteins, and on ways in which this can be done systematically and purposefully, within the framework of a working hypothesis where redox side-reactions hold an important role in the physiological outcome of experimental transfusions with artificial oxygen carriers.

## 1. Introduction: Structure and Reactivity of Hemoglobin

**Figure 1:**
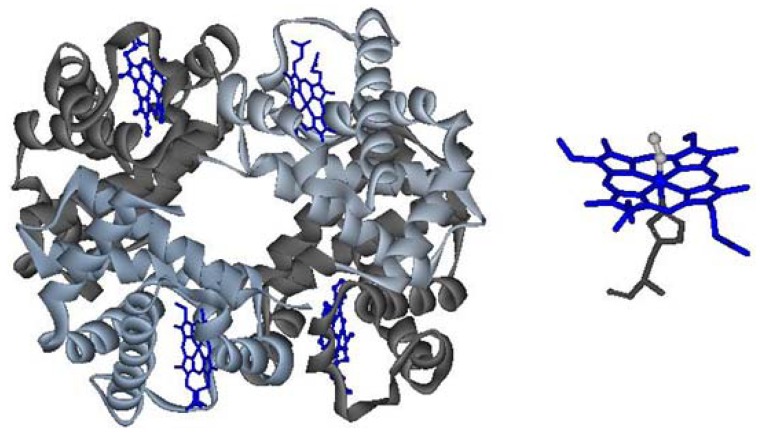
Left: quaternary structure of hemoglobin; right: heme, in its oxy state.

Hemoglobin (Hb) is the protein responsible for the transport of oxygen from the lungs to the other tissues of the body and participates in the transport of carbon dioxide in the opposite direction. Hb is a metalloprotein whose structure consists of four monomeric units: two α and two β chains, held together by noncovalent interactions - hydrogen bonds and salt bridges. Each monomer contains a heme *b* group whose iron atom binds molecular oxygen; *trans* to the oxygen coordination position the iron is bound to the polypeptide via the so-called “proximal histidine” (Figure 1) [1,2].

Transport of oxygen by hemoglobin is achieved by alternating the structure of two forms: the high oxygen affinity relaxed (R) state oxyhemoglobin, *vs*. the low oxygen affinity tense (T) state deoxyhemoglobin. Allosteric effectors such as 2,3 BPG modulate this reactivity. The negatively charged BPG is inserted into the central cavity between the two β chains of deoxy molecule, next to the positively charged His2, Lys82 and His143 [1,3]. Oxygen binding in hemoglobin is cooperative, with the binding of an O_2_ molecule facilitating the binding of further oxygen to the remaining subunits. Oxygen binding and conversion to the R state cause conformational changes that involving the rotation of one dimer relative to the other. These changes in turn cause reduction of the space allocated for the BPG binding site, thus forcing it to leave the Hb molecule. In addition to BPG, other factors such as H^+^, NO, ATP, Cl^−^, or CO_2_ modulate the oxygen affinity [3,4]. Bovine Hb (bHb) is not as dependant as human Hb on the effects of BPG, thus providing an advantage towards simplicity for bHb in preparation of blood substitites [4]. Additionally, other sources of Hb are also considered, including more exotic ones such as *Lumbricus*, arctic fish and others [5,6].

To bind and transport oxygen, the iron must be in the ferrous (Fe^2+^) state. On the other hand, as a side-reaction, oxyhemoglobin also undergoes spontaneous oxidation (autooxidation) of the iron, forming metHb (Fe^3+^); this latter state cannot bind oxygen [7]:

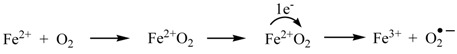



Overall, as an effect of the autooxidation reaction reactive oxygen species (ROS) such as hydrogen peroxide and free radicals (especially superoxide, but also hydroxyl radical and hydrogen peroxide generated upon degradation of superoxide) are generated, with damaging effects on organs and living cells. Methemoglobin reductase is the enzyme within the erythrocyte that acts to reduce the met form back to deoxyHb [8]. A separate range of reactions takes Hb onto nitrosative stress routes [6].

## 2. Reaction of Peroxide with the Heme Group

The heme group is intrinsically reactive towards hydrogen peroxide, typically engaging in a two-electron reduction of H_2_O_2_ to H_2_O. The reaction of hydrogen peroxide with oxyHb results in the oxidation of the Hb to Fe^4+^, with two electrons moving from iron to H_2_O_2_. The reaction with metHb results in the formation of Fe^4+^, with a second electron coming from the globin and hence leading to free radical formation (R, where “R” may be an aminoacid from within the hemoglobin or, if available, an external reducing agent such as ascorbate, urate, phenolics, and others) [9,10,11,12,13]:

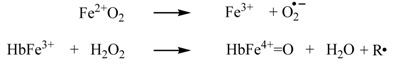



Hydrogen peroxide thus activates hemoglobin, leading to high valent ferryl (Fe^4+^-oxo) intermediates at the heme iron—Compound I (ferryl plus radical) and Compound II (ferryl). Ferryl Hb is constantly produced in the body and in condition of stress, illness or high-intensity exercise; it acts as a radical-generating agent, inducing, among others, the peroxidation of lipids [8].

Cell-free hemoglobin is lethal, because of extravasation, redox reactivity, kidney filtration, oxygen affinity, NO dioxygenase reactivity [14,15,16,17]. However, purified hemoglobin can be a reasonable blood substitute candidate once its reactivities can be controlled and/or reduced. To this end, a series of chemical modifications/transformations were performed as follows/as further described (with main directions summarized in Table 1).

**Table 1:** Options for hemoglobin modification.

Modified Hb forms	
Intramolecular crosslinking	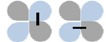
Intermolecular crosslinking	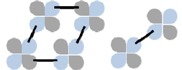
Single step derivatization	
Pegylation	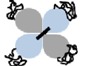
Genetically modify hemoglobin Tyr ↔ Val, Trp / Phe ↔Tyr	[possibly combined with any of the above]
The encapsulation of hemoglobin	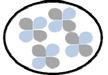

## 3. Intramolecular Crosslinking

A first approach for containment of hemoglobin’s reactivity involves covalent crosslinking of the monomers so as to avoid tetramer dissociation. This method was extensively practiced in order to suppress renal filtration by preventing tetramer dissociation [14,15]. Reagents like acetaldehyde, formaldehyde, bis(3,5-dibromosalicyl) fumarate (DBBF) and nor-2-formylpyridoxal 5-phosphate (NFPLP) or pyridoxal phosphate have potential in this respect—in some cases for multiple intramolecular crosslinking interactions.

**Scheme 1:**
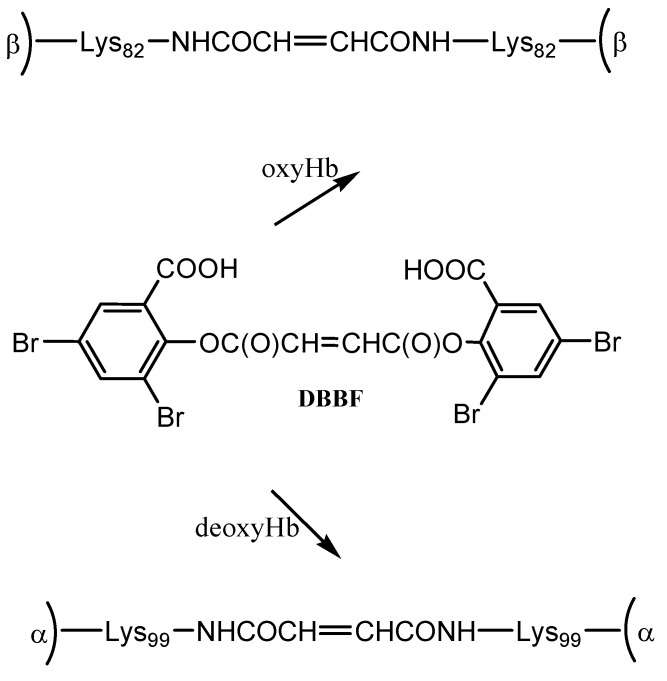
Reaction of hemoglobin with DBBF.

DBBF is a negatively charged reagent which upon reaction with oxyHb produces crosslinks between the two β chains. The resulting product has a higher affinity for oxygen than native Hb. When performed on deoxyHb, acylation with DBBF occurs between the Lys99 of two α chains (Scheme 1). This reaction occurs in the presence of tripolyphosphate, which acts to block competitive sites at the Lys-82β and Val-1β. The product obtained has oxygen affinity substantially reduced [15]. DBBF-Hb’s side effects connected most likely with prooxidant reactivity [18] have reduced its usefulness as a blood substitute.

**Scheme 2:**
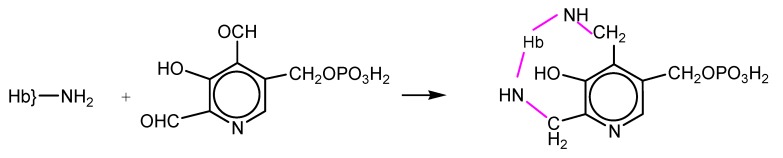
Reaction of hemoglobin with NFPLP.

NFPLP contains two reactive aldehyde groups and crosslinks deoxyHb at two sites: at the N-terminal amino group of one β subunit and at Lys82 of the other (Scheme 2). The oxygen affinity was decreased but the rate of autooxidation was increased. The dissociation into αβ dimers was prevented, the elimination of Hb in the urine was almost completely prevented and accumulation in the kidneys was also diminished [15,16].

*Intermolecular crosslinking (polymerization) of hemoglobin* was employed in order to generate particles of large molecular weight/volume, which would then display increased stability in the bloodstream and avoid homogenous close contact between hemoglobin and endothelium, thereby reducing the hemoglobin-NO reactivity [15]. Most useful reagents achieve both intra- and intermolecular crosslinking simultaneously, in a proportion that depends on the concentration of the reactants, on the iron state, reaction time, etc. The reaction often occurs through a Schiff base formation between the aldehyde ends of the reagent and the amine moieties of the protein [8,15].

**Scheme 3:**
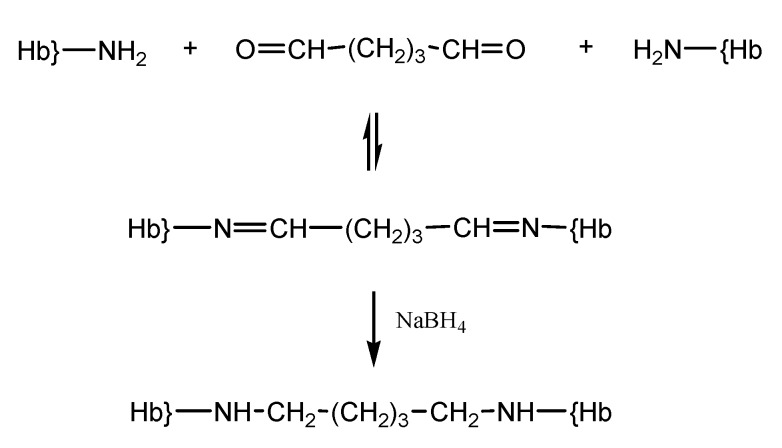
Reaction of hemoglobin with glutaraldehyde.

Glutaraldehyde is a bifunctional reagent most frequently utilized for polymerization of Hb, in the context of its wide applicability in biochemistry as a crosslinker [19]. Hemoglobin derivatization with glutaraldehyde allows intermolecular bonding between the amino groups of lysines and valines on Hb and the carbonyl groups of glutaraldehyde (Scheme 3). This reaction yields chemically unstable imines, which can be easily hydrolyzed in aqueous solution yielding the starting glutaraldehyde cross-linker and free hemoglobin. Therefore, to avoid regeneration of the amino and carbonyl functions, the reducing agent NaBH4, or derivatives thereof, are used to reduce the imine bonds into stable amine bonds [19,20].

**Figure 2:**
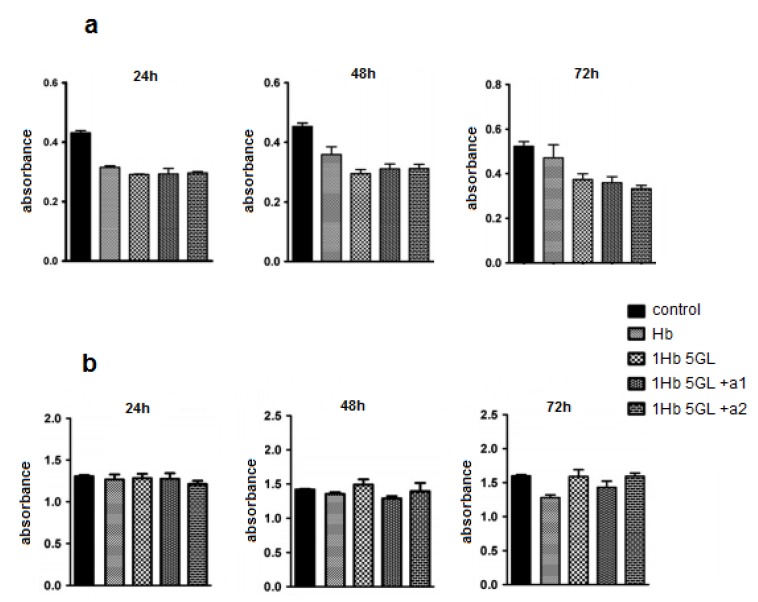
Effect of hemoglobin derivatization with glutaraldehyde on HUVEC cultures (**a**) and human lymphocytes (**b**) after 24, 48 and 72 h treatments.

Our published results show that using such protocols polymers with molecular weight > 500 kDa can be obtained. The peroxide and ascorbate affinity for the polymerized product were measured and compared with that of native Hb; the calculated Michaelis-Menten constants showed that polymerized product has low affinity for hydrogen peroxide but high affinity for ascorbic acid. The oxygen affinity was also higher than native Hb [19]. Test results obtained from HUVEC cells (Figure 2a) indicate that native hemoglobin as well as poly-Hb display a slight inhibitory effect on HUVEC cultures. It should be noted that addition of antioxidants like ascorbic acid, BSA, catalase, glucose, to poly-Hb does not appear to improve the performance to any significant extent. Tests in human lymphocytes (Figure 2b) show that after 24 h, Hb compounds did not inhibit significantly the lymphocyte viability [21]. Hemoglobin derivatives show a slightly inhibitory effect after 48 h, but not statistically significant. At 72 h a slight difference also begun to develop between native and polymerized Hb, in favor of the polymerized protein [21].

It was also shown possible to produce a polymerized Hb with low oxygen affinity (P_50_~25–30 mmHg) by mixing a diluted solution of Hb with glutaraldehyde. Only ~28% of the product contained polymer with molecular weight > 500 kDa. Test in rats showed a retention time of 15 h in plasma while tests in pigs showed no renal toxicity, though mean arterial pressure and pulmonary arterial pressure increased in a hemorrhagic shock [15,22].

A high degree of polymerization (>95%) (MW: 130–500 kDa) and low affinity for oxygen was obtained in the case of the glutaraldehyde-polymerized bovine hemoglobin—product called *Hemopure* produced by the Biopure Coporation (currently OPK Biotech LLC, Cambridge, MA, USA). The product progressed to Phase II clinical trials [15] and was approved for limited human use in South Africa, in a context where HIV and other issues put significant limits on the availability of donated human blood [8,23]. Nevertheless, neither this not other Hb-based blood substitute is approved for human use anywhere else in the world.

**Scheme 4:**
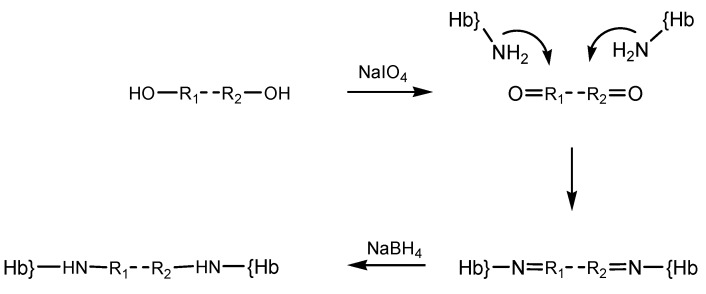
Step derivatization of hemoglobin with periodate-generated reticulation agents.

Other reticulating agents include periodate-generated ones. Periodate modification of the diol moieties in sugars had previously been employed in order to prepare dialdehyde-type reagents, which were then utilized in crosslinking reactions on hemoglobin, yielding polymerized material with useful dioxygen-binding properties and hence proposed as possible artificial oxygen carriers [15,24]. The periodate protocol was shown to be applicable to a wider range of oxygen-containing compounds, illustrated by starch, polyethylene glycol [25] and alginate. Derivatization reaction steps with such reagent whose carbonyl groups were generated by oxidation with NaIO_4_ is illustrated in the diagram below (Scheme 4). For simplicity the reagent was noted OH-R_1_-R_2_-OH.

The dioxygen-binding properties and redox reactivities were investigated for the derivatized hemoglobins, with emphasis on pro-oxidative properties. The published results indicate that there is a general tendency of the derivatization to result in higher autooxidation rates, especially for those obtained with polyethylene glycol [25]. The anionic character of the periodate and of its byproducts does raise the issue as to whether part of these increases in prooxidant reactivity might be due to remnant iodine-derived redox-active anions on the Hb; though in our experience extensive washing and/or column purification of the derivatized Hb should remove any such anions, the issue is not entirely closed [26].

**Figure 3:**
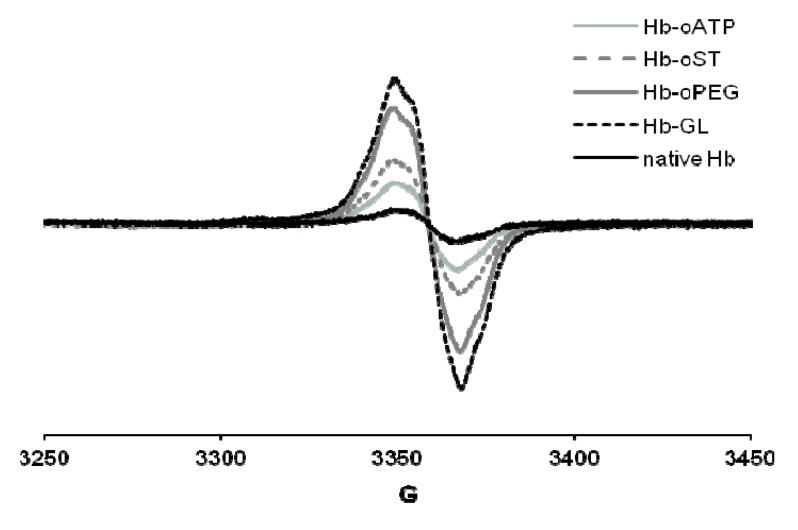
EPR spectra of globins treated with H_2_O_2_. Conditions: 200 µM protein, 400 µM hydrogen peroxide, PBS, frozen at 30 s after mixing. Hb-oATP: Hb derivatized with periodate-oxidized ATP; Hb-oST: Hb derivatized with periodate-oxidized starch; Hb-oPEG: Hb derivatized with oxidized PEG; Hb-GL: glutaraldehyde-polymerized Hb.

The peroxide reactivity of the met (ferric) form was also affected by derivatization, as witnessed, among others, by varying yields of ferryl (Fe(IV)-oxo) and free radical generated. In cell culture tests (human umbilical vein epithelial cells, HUVEC), the derivatization protocols showed no toxic effect. All the derivatization protocols described in [25] led to up to 10 times more free radical—the maximum yield being reached in glutaraldehyde-derivatized Hb, followed closely by oPEG-Hb (Figure 3). On the other hand, scaled superposition of the signals confirms that the shape of the free radical signal is not different in the five proteins examined – which, among others, suggests that the adenine ring in oATP-Hb is not a stable site for free radicals at least not by comparison with tyrosine residues within Hb, which dominate our EPR spectra [25]. However this reaction has been studied in early 1980s. Open ring ATP was used by different groups. While Greenburg and Maffuid [27] reported a high autooxidation rate of oATP-Hb, Hsia [28] did not report such a problem. Like in case of Greenburg’s group, it is very probable that our reaction volume is contaminated with IO_3_/IO_4_ from the reaction of ATP with NaIO_4_.

## 4. Single-step Hemoglobin Derivatization

There are some protocols for derivatization of hemoglobin with two N-hydroxysuccinimide esters (NHS-esters): disuccinimidyl suberate (DSS) and (methyl-PEG_12_)_3_-PEG_4_-N-hydroxysuccinimide ester (TMS). They allow protein crosslinking without toxic side-products and forming peptide bond with lysine residue in one single step [29].

**Figure 4:**
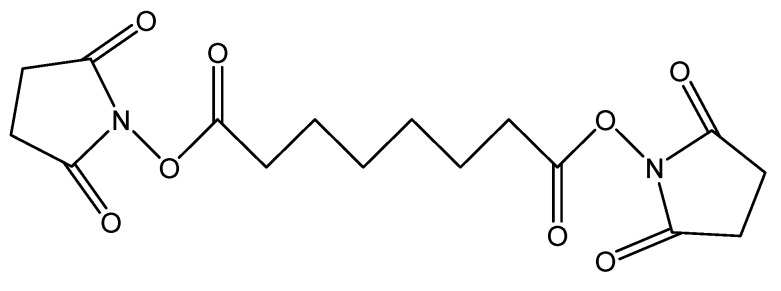
Chemical structure of DSS.

The first agents, DSS (Figure 4), is a non-cleavable membrane permeable crosslinker that contains an amine-reactive N-hydroxysuccinimide ester at each end of an 8-carbon spacer which reacts with lysine residues to form peptide bond in one single step [29].

DSS may be employed for obtaining Hb polymers, and the increase in autooxidation rate incurred by this polymerization is completely reversed when BSA is co-polymerized with Hb. The copolymers show very little change in autooxidation and oxygen affinity compared to native Hb, and low intensity of free radicals – less than in native Hb [29].

A third approach towards reducing heomglobin’s pro-oxidant activity has involved *derivatization of the protein surface with polymeric systems*. The polymers have the requirement to be non-toxic, non-immunogenic, non-antigenic, and soluble in water; such are polyethylene glycol, and some oligo- and polysaccharides. This method has similar results to those based on polymerization, as the aim is again to increase molecular weight, reduce toxicity, and prevent renal excretion. 

Typically, the first step in pegylation is suitable derivatization of the PEG at one or both ends, based on the type of available reactive group on protein. The aminoacids most frequently involved in pegylation are lysine and cysteine [15]. If only one end of the PEG is reactive, the Hb molecule is simply decorated with PEG strands - but there are several possibilities of intra- and intermolecular cross-linking if both ends of the reagent are functional. In the second case, a large part of research was focused of the pyridoxalated Hb where, contrary to glutaraldehyde-polymerized pyridoxalated Hb, cooperativity was conserved. In both cases the size of the protein is increased. [15,30,31].

**Figure 5:**
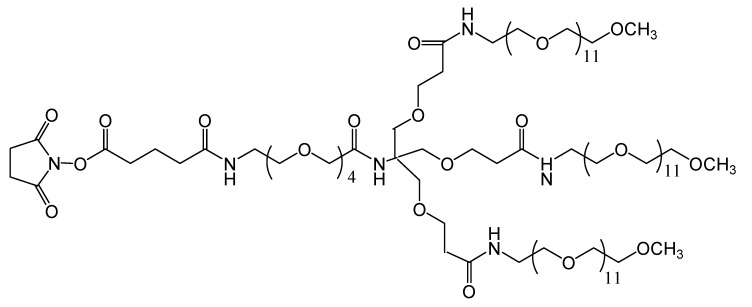
Chemical structure of the amine-reactive PEGylation reagent used (methyl-PEG_12_)_3_-PEG_4_-N-hydroxysuccinimide ester).

TMS (Figure 5), is a branched amine-reactive PEGylation reagent (methyl-PEG_12_)_3_-PEG_4_-N-hydroxysuccinimide ester. Each methyl-terminated PEG (mPEG) branch contains 12 ethylene glycol units. The three branches are attached to a 4-unit PEG stem that contains an amine-reactive *N*-hydroxysuccinimide (NHS) ester at the distal end [30].

Our published results show that rate of autooxidation during the process of pegylation with TMS is very low, and that the dioxygen affinity of this derivatized Hb is very close to that of native bovine Hb, with the particular observation that, unlike with most other derivatization protocols [30]. In cell culture tests (human umbilical vein epithelial cells, HUVEC), the derivatization protocol induces no toxic effect. These results show promise towards applicability for production of hemoglobin-based blood substitutes [30].

A fourth approach has been to *genetically modify hemoglobin*. Genetic engineering offers an alternative to chemically modified Hb. Modifications have been made to increase the tetramer’s stability, decreased its oxygen affinity and to reduce the reactivity towards NO. The latter inconvenient can be dealt with by modifying amino acids gating access of small molecules towards the heme site [8,15,16,32]. Examples of recombinant hemoglobin include rHb1.0, rHb1.1, rHb1.2—proteins that contain the Presbyterian mutation and have zero, one or two glycines linking the alpha subunits, respectively [16].

Recombinant cross-linked Hb (rHb1.1, *Optro*) is a variant of human Hb, expressed in *E. coli* and developed for commercialization. This hemoglobin contains two modification: the first modification fuses the alpha subunits: the C-terminal arginine of one α chain and the N-terminal valine of the other are genetically linked by a glycine bridge [33]. The other modification contains the Presbyterian mutation and involves the change of Asn108 →Lys in both β-chains. This mutation decreases the oxygen affinity to a level comparable to that of native one [33,34].

Hemoglobin Polytaur is an autopolymerizing human-bovine hybrid mutant and was obtained by polymerization of Hb Minotaur—a hybrid that contains α-human and β-bovine subunits [15]. The reaction of polymerization occurs via the oxidation of thiol groups (-R-SH SH-R)→ -R-S-S-R) between Cys residues introduced at the external position β9. Two forms were obtained: Hb Polytaur (Cys104α→Ser, Cys93β→Ala) with MW: 500 kDa and (Hb Polytaur)_n_ with MW 1,000 kDa or higher. The polymer designated as Hb Polytaur has an oxygen affinity similar with Hb-A but the rate of autooxidation is increases [15,35,36].

*The encapsulation of hemoglobin in liposomes or vesicles* is another approach that can influence hemoglobin’s reactivity [8]. The encapsulation of natural or modified Hb in an artificial membrane is expected to lead to fewer side effects, longer intravascular duration and greater oxygen-carrying capacity [16].

**Figure 6:**
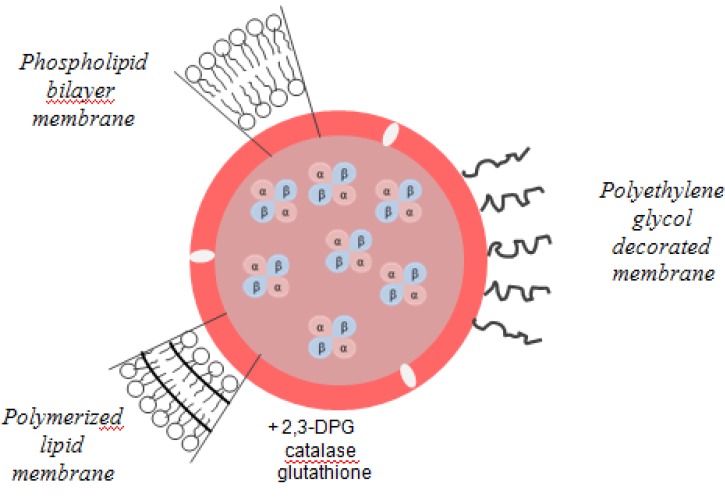
Liposome-encapsulated hemoglobin.

Liposomes are closed vesicles of lipid-bilayer membrane and are the most investigated system because of their higher biocompatibility and biodegradability and their capacity to increase the retention time of entrapped agents inside the cell [14]. The lipid composition of such vesicles is based on a mixture of saturated phospholipids with a large number of carbon atoms like distearoylphosphatidylcholine or dipalmitoylphosphatidylcholine and cholesterol, which reduces membrane permeability prevents hemoglobin oxidation and denaturation, and reduces lipid peroxidation [14,22]. The size of liposome and the encapsulation efficiency are decisive factors regarding their use. Larger sized liposomes are rapidly removed from circulation by reticuloendothelial system (RES). Another disadvantage is the extensive oxidation of the protein during encapsulation and storage. In this case it is essential to co-encapsulate antioxidants such as catalase, methemoglobin reductase, superoxide dismutase, and glutathione. The oxygen affinity can also be modified to be close to that of RBCs, by co-encapsulation of allosteric effectors such as 2,3-BPG, inositolphosphate or pyridoxal phosphate. Co-encapsulation with the latter effector generates a product with an oxygen affinity similar to the red blood cell [22,37,38,39]. Liposome-encapsulated hemoglobin (LEH) has been modified to achieve stability in circulating blood. Thus, derivatives of polyethylene glycol, polysaccharides and phosphatidylinositol were added to this product in order to modify the surface of liposomes and to thereby decrease reticuloendothelial system uptake and prolong LEH circulation persistence. Surface modification with PEG appeared to be the most promising strategy for increasing the circulation time (65 h). The stability of the bilayer membranes was also increased by polymerization, with a 4-fold increase in plasma (Figure 6) [16,40].

Liposome-encapsulated Hb was first produced in the United States. Later it became the subject and the target of Japanese researchers, who have dubbed the term hemoglobin vesicles (HbV). As in the case of liposomes, the Hb solution is encapsulated within a phospholipid bilayer membrane. A particle has a diameter of ~250 nm and contains about 30,000 Hb molecules. One of the properties that can influence the phospholipid bilayer is the fluidity and temperature [16]. Thus, the number of bilayers decreases when the surface potential of the lipid membrane becomes more negative and membrane fluidity is reduced inversely with the temperature. The encapsulation efficiency, oxygen affinity and the rate of metHb formation are sensitive to pH, the optimum being 7 to 7.4 [16].

Tests on animals showed an efficient transport of oxygen, comparable with the RBCs; the product has not yet been tested on humans [15,16].

A sixth approach involves supplementation with *small-molecule antioxidants* (*e.g*., ascorbate or selenium). The aim of this method is to provide an oxygen carrier with antioxidant properties. In order to reduce the autooxidation rate and prooxidant reactivity, various antioxidants can be introduced into the reaction mixture when derivatizing hemoglobin, under conditions where the dioxygen affinity and Hill coefficient are maintained at levels close to that of native hemoglobin (e.g., as seen in Table 2) [25]. These features are particularly useful since chemical derivatization of hemoglobin, especially in the form of crosslinking, can lead to autooxidation, increased affinity, and decreased cooperativity. Also, copolymerization with BSA lowers the amount of free radicals generated upon reaction of hemoglobin with hydrogen peroxid. This may be expected to be a positive feature in a blood substitute [25,41].

**Table 2:** Autooxidation rates and P_50_ values expressed in percentage increases relative to nHb, and Hill coefficients for Hb-albumin copolymers [25].

	Autooxidation	P_50_	Hill coefficients
Hb-oATP	+110%	−66%	1.99
Hb-oATP-1 mM BSA	−33%	−55%	1.97
Hb-oATP-2 mM BSA	−33%	−36%	1.79

## 5. Hemerythrin-Based Blood Substitutes

Hemerythrin-based blood substitutes use as active ingredient a protein responsible for oxygen transport in the marine invertebrates that employs a non-heme diiron active site (Fe(II)-Fe(II)). Hemerythrin (Hr) was shown to avoid reactivity towards hydrogen peroxide, nitric oxide and nitrite. Another advantage is the higher molecular weight than Hb (108 kDa vs 64 kDa), which should lead to lower levels of extravasation or elimination through the kidney [42,43]. Until now, glutaraldehyde-polymerized hemerythrin and polyethylene glycol-derivatized hemerythrin were successfully obtained. The effects of these chemical modifications on molecular weight, autooxidation rate and oxygen affinity appear to be favorable for blood substitute applications [42,43,44,45].

The toxicity of hemerythrin and of its chemical derivates was tested on cultures of human cells, comparing their performance with that of the representative competitor, glutaraldehyde-polymerized bovine hemoglobin [20]. Hemerythrin (native or derivatized) exhibits a proliferative effect on HUVEC cultures, as opposed to a slight inhibitory effect of Hb. A similar positive effect is displayed on human lymphocytes by glutaraldehyde-polymerized hemerythrin, but not by native or polyethylene glycol-derivatized hemerythrin. Thus, hemerythrin and its chemical derivatives were found to be less toxic than native Hb and glutaraldehyde-polymerized Hb [20].

To conclude, there is now available a library of potential blood substitutes with varying degrees of reactivity towards oxidative stress agents. For several of these, there appears to be some degree of positive correlation between lack of reactivity in chemical versus cell culture tests. Ongoing tests on animal systems are hoped to confirm that rational design of a protein-based blood substitute is within reach.
